# Characteristic target sign of the small intestine in a case of lupus enteritis

**DOI:** 10.1002/jgf2.517

**Published:** 2021-12-29

**Authors:** Toshimasa Yamaguchi

**Affiliations:** ^1^ Primary Care and Advanced Triage Section Osaka City General Hospital Osaka Japan

**Keywords:** imaging, intestinal tract, lupus enteritis, systemic lupus erythematosus, target sign

## Abstract

A 30‐year‐old woman was admitted to our hospital complaining of intermittent severe abdominal pain. Her laboratory examinations revealed positive serology for antinuclear antibody titer of 1:640 (reference < 40), double‐stranded DNA antibody, anti‐Smith antibody, and anti‐RNP antibody. A contrast‐enhanced computed tomography demonstrated slight ascites and a characteristic target sign, suggesting lupus enteritis.

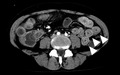

A 30‐year‐old woman, who complained of intermittent severe abdominal pain and nausea, was admitted to our hospital. She had been admitted to a referral hospital for the same complaint 6 months previously, where a plain abdominal computed tomography scan had revealed ascites and slight thickening of the small intestinal wall. Her severe abdominal pain had persisted and an exploratory laparotomy was performed; however, the etiology of her symptoms was not identified. The abdominal pain was thought to be caused by an allergic reaction and autoimmune disease. She was treated with a course of steroids for idiopathic thrombocytopenia. The abdominal pain subsided spontaneously, and she was not tested for antinuclear antibodies.

On admission, she was conscious, and her body temperature, blood pressure, and pulse rate were 36.9°C, 136/60 mmHg, and 70 beats/minute, respectively. Further physical examination showed tenderness across the lower abdomen, without guarding, and edema of the face and lower legs. No malar rash, oral ulcers, alopecia, Raynaud’s phenomenon, or arthritis was observed. Laboratory tests revealed the following: white blood cell count, 8.84 × 10^9^ cells/L (reference, 3.17–8.40 × 10^9^ cells/L); hemoglobin, 140 g/L (reference, 110–147 g/L); platelets, 88 × 10^9^/L (reference, 125–375 × 10^9^/L); and creatinine level, 44.2 μmol/L (reference, 35.4–70.7 μmol/L). A urine test revealed proteinuria.

Contrast‐enhanced computed tomography revealed mild ascites and a characteristic target sign (Figure 1) due to jejunal wall thickening, with inner and outer circumferential enhancement and attenuation of the middle layer. Additional laboratory tests revealed significantly low C3 and C4 complement levels of 0.39 g/L (reference, 0.80–1.60 g/L) and 0.06 g/L (reference, 0.17–0.45 g/L) respectively; positive serology for antinuclear antibodies with a titer of 1:640 (reference, <40); a double‐stranded DNA antibody titer of 73.0 IU/ml (reference, <10 IU/ml); an anti‐Smith antibody titer of 25.1 U/ml (reference, <7.0 U/ml); and an anti‐RNP antibody titer of 65.2 U/ml (reference, <10.0 U/ml). Based on these findings, the patient was diagnosed with systemic lupus erythematosus (SLE)‐associated enteritis according to the 2019 European League Against Rheumatism and American College of Rheumatology classification criteria for SLE.^1^

**FIGURE 1:**
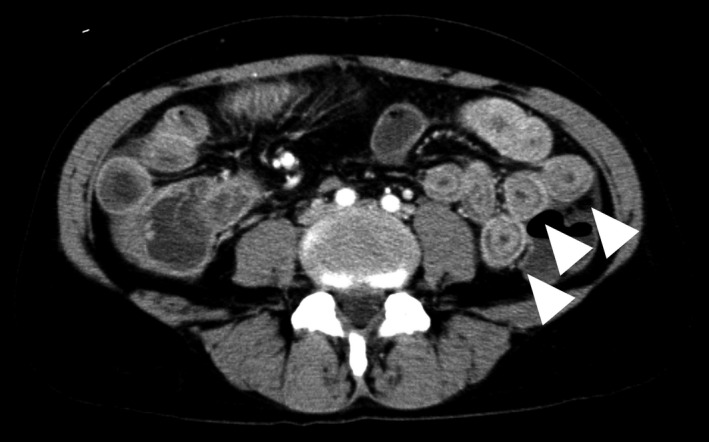
Abdominal contrast‐enhanced computed tomography demonstrating the edematous and thickened small intestinal wall with inner and outer circumferential enhancement known as the target sign (white arrowheads)

The patient was administered a 3‐day course of intravenous methylprednisolone pulse therapy, followed by prednisolone 1 mg/kg/day. However, her abdominal pain, hypocomplementemia, and proteinuria persisted. Four weeks after starting steroid therapy, a renal biopsy was performed, which revealed diffuse proliferative lupus nephritis, accompanied by vasculitis in the preglomerular arterioles. Therefore, the patient was treated with oral cyclophosphamide, which relieved the symptoms, and the prednisolone dosage was tapered every 2 weeks. The patient was discharged after 4 weeks. After 6 weeks of combined cyclophosphamide and corticosteroid therapy, a renal biopsy revealed that the nephritis had improved; however, the hypocomplementemia persisted.

Lupus enteritis includes lupus mesenteric vasculitis, which is the most common gastrointestinal manifestation associated with SLE; intestinal pseudo‐obstruction; protein‐losing enteropathy; and thrombosis of the vasculature.^2^ The small intestine is most commonly affected in lupus mesenteric vasculitis. The histopathology of this condition reveals immune complex deposition and complement activation with subsequent submucosal edema which causes edema and thickening of the small intestinal wall, leukocytoclastic vasculitis, and thrombus formation.^3^ Moreover, the target sign of the intestinal tract on contrast‐enhanced computed tomography, which reflects the histopathological features of this condition, is one of the most useful findings for diagnosing lupus enteritis.^4^

The differential diagnosis of edematous and thickened small intestinal walls includes Henoch‐Schönlein purpura, intestinal anisakiasis, and eosinophilic gastroenteritis. Therefore, appropriate diagnosis of lupus enteritis requires a comprehensive evaluation including imaging, as well as consideration of the clinical and serological findings. Lupus enteritis is relatively rare and is only present in 0.2–9.7% of cases of SLE.^3^ Lupus enteritis is sometimes mistakenly diagnosed as an acute abdomen requiring emergency surgery because lupus mesenteric vasculitis often presents with sudden onset of intense abdominal pain.^3^ Furthermore, lupus enteritis can develop as the initial or the only active presentation of SLE.^5^ In our patient, the presence of thrombocytopenia and proteinuria, which are potential complications of SLE, enabled us to interpret the patient's abdominal computed tomography findings as suggestive of lupus enteritis.

If clinicians consider lupus enteritis in the differential diagnosis of enteritis, the characteristic imaging findings are helpful for early diagnosis, especially in young and middle‐aged women, enabling appropriate therapeutic intervention.

## CONFLICT OF INTEREST

The authors declare no conflict of interests for this article.

## PATIENT CONSENT FOR PUBLICATION

Obtained.
